# Promoter polymorphisms of *DNMT3B *and the risk of colorectal cancer in Chinese: a case-control study

**DOI:** 10.1186/1756-9966-27-24

**Published:** 2008-07-28

**Authors:** Hong Fan, Feng Zhang, Jiabo Hu, Dongsheng Liu, Zhujiang Zhao

**Affiliations:** 1 Key Laboratory of Developmental genes and Human diseases, Ministry of Education, Southeast University, 210009, Nanjing, PR China; 2 Department of Genetics & Developmental Biology, The School of Basic Medical Sciences, Southeast University, 210009, Nanjing, PR China; 3 School of Medicine, Jiangsu University, 212001, Zhenjiang, PR China

## Abstract

**Background:**

DNA-methyltransferase-3B (DNMT3B), which plays a role in DNA methylation, is usually aberrant expression involved in carcinogenesis. Polymorphisms of the DNMT3B gene may influence DNMT3B activity on DNA methylation in several cancers, thereby modulating the susceptibility to cancer.

**Methods:**

DNMT3B -579G>T genotypes and -149C>T were determined by PCR-RFLP and sequencing in 137 colorectal cancer patients and 308 controls matched for age and sex, who did not receive radiotherapy or chemotherapy for newly diagnosed and histopathologically confirmed colorectal cancer. The association between two SNPs of the *DNMT3B *promoter and the risk of the development of colorectal cancer was analyzed in a population of Chinese.

**Results:**

The allele frequency of -149C >T among patients and controls was 0.73% versus 0.65%, respectively. The allele frequency of -597G>T for patients and controls was 6.57% versus 11.53%, respectively. Individuals with at least one -149C>T allele were no at a significantly increase risk of colorectal cancer compared with those having a -149TT genotype. However, Individuals with at least one 579G>T allele were decreased risk of colorectal cancer compared with those having a -579TT genotype.

**Conclusion:**

The relative distribution of -149C>T *DNMT3B *SNPs among a Chinese population can not be used as a stratification marker to predict an individual's susceptibility to colorectal cancer. However, the DNMT3B -579G>T polymorphism may contribute to the genetic susceptibility to colorectal cancer.

## Background

Single nucleotide polymorphisms (SNPs) are the most common form of human genetic variation, and may contribute to an individuals' susceptibility to cancer. Some studies have suggested that some variants in the promoter regions of genes may affect either the expression or activity levels of enzymes [1-3] and therefore may be mechanistically associated with cancer risk. DNA methylation is a major epigenetic modification involving the addition of a methyl group to the 5' position of a cytosine in a CpG dinucleotide. A number of studies have suggested that aberrant DNA cytosine methylation may play an important role in carcinogenesis [4-8]. DNMT3a and DNMT3b are required for the establishment and maintenance of genomic methylation patterns and proper murine development [9-12]. Both genes are up-regulated to differing degrees in some malignancies, including esophagus carcinoma, lung cancer and colorectal cancer [13-18]. Recently, several studies showed that some of SNPs in the *DNMT3B *gene may influence *DNMT3B *activity on DNA methylation, thereby modulating the susceptibility to lung cancer, breast cancer and gastric cardiac adenocarcinoma [14,19,20]. The *DNMT3B *gene contains a single C>T transition polymorphism (C46359T, GenBank accession no. AL035071) in the promoter region of the *DNMT3B *gene, -149 base pairs from the transcription start site, is reported to greatly increase promoter activity [21]. Some reports have shown that the C/T polymorphism is associated with an increased risk for lung cancer and carcinoma of the head and neck. Carriers of T allele, particularly heterozygotes, have a significantly increased risk for such cancers[2,22-24]. However, C/T polymorphism is not associated with the increased risk of hapetocellular carcinoma (HCC) and gastric cancer, especially in Chinese. Another G>T SNP of *DNMT3B *gene, in the transcription start site of the promoter region (-579 bp from exon 1B, GenBank accession no. NT_028392) and this probably affects gene function [2]. Some studies have suggested that the *DNMT3B *-579 G>T may modify susceptibility to tumors, although conflicting results have been reported in different tumor types, the heterozygous genotype have been reported to have a significantly reduced risk of developing lung and colorectal cancer [25-27], but *DNMT3B *genetic polymorphism is variable in different races, ethnic groups or geographic areas. In the present study, we evaluated the association of _149C>T and _579G>T polymorphisms with colorectal cancer in Chinese population.

## Methods

### Study population

This case-control study included 137 colorectal cancer and 308 healthy controls, and the informed consent was obtained. The 137 case subjects were patients who had undergone surgery and been histopathologically confirmed at the Zhongda Hospital of Southeast University and Tumor Hospital, Nanjing, China. The control subjects were selected from a pool of cancer-free subjects who visited the same hospital for a regular physical examination and who volunteered to join the epidemiology survey during the same period. We defined a healthy subject as a person free of disease (including no history of cancer) on health check-up. They were matched with the case patients by age and sex (Table 1). All cases and controls were ethnically Chinese and resided in Jiangsu province or in the surrounding regions.

**Table 1:** Characteristics of the study population.

Variables	Case (n = 137)	Control (n = 308)
Ages (years)*	65	71
Sex		
Male	91	206
Female	46	102

* *p *> 0.05

### DNA extraction

5 milliliters of venous blood from each subject was drawn in vacuum tubes containing EDTA and stored at 4°C. Genomic DNA was extracted within one week after sampling by using proteinase K digestion followed by a salting out procedure [28].

### DNMT 3B genotyping

The transition of C/T of *DNMT3B *SNP creates a *BlnI *restriction site and the transition from G to T of the *DNMT3B *SNP creates a *PvuII *restriction site, which could be exploited for genotyping by PCR and subsequent restriction fragment length polymorphism (RFLP) analysis. PCR was performed in a volume of 25 μL containing 100 ng of DNA template, 10 × PCR master mix (Promega, USA), and 10 pmol/L each of sense primer (5'-TGCTGTGACAGGCAGA2GCAG-3') and antisense primer (5'-GGTAGCCGGGAACTCCACGG-3') for _149C>T, and sense primer (5'-GAGGTCTCATTATGCCTAGG-3') and antisense primer (5'-GGGAGCTCACCTTCTAGAAA-3') for _579G>T. For PCR amplification, an initial denaturation step at 94°C for 5 min was followed by 30 cycles at 94°C for 30 s, 57°C for 30 s, and 72°C for 30 s, and a final extension step at 72°C for 7 min. Subsequently, the PCR products were digested overnight with *Blnl *(TaKaRa) 4 U for 149C>T and 5 units of *PvuII *(New England Biolabs, Beverly, Mass.) for 579G>T at 37°C, respectively, and the products separated on 2% agarose gels. RFLP bands were visualized by ethidium bromide staining under UV light. The 149C>T polymorphism depend upon the existence of the *Blnl *recognizing site, the *DNMT3B *T/T genotype was expected to show two DNA bands at the positions of 207 and 173 bp, the C/C genotype was expected to show a single band (380 bp), while the heterozygote was expected to have three bands (380, 207 and 173 bp). The 579G>T polymorphism was determined by *Pvu*II. The wild-type G allele has only one band, while the polymorphic T allele has two bands (132 and 93 bp). For quality control, genotyping analysis was performed blind with respect to case/control status and repeated twice for all subjects. The results of genotyping were 100% concordant. To confirm the genotyping results, selected PCR-amplified DNA samples (n = 3, respectively, for each genotype) were examined by DNA sequencing, and the results were also 100% concordant.

### Statistical analysis

Cases and controls were compared using Student's t-test for continuous variables and the X^2 ^test for categorical variables. Hardy – Weinberg equilibrium was tested with a goodness-of-fit X^2 ^test with one degree of freedom to compare the observed genotype frequencies with the expected genotype frequencies among the subjects. Comparison of the *DNMT3B *genotype and allelotype distribution in the study groups was performed by means of two-sided contingency tables using X^2 ^test or Fischer's exact test. The odds ratio (OR) and 95% confidence interval (CI) were calculated using an unconditional logistic regression model and adjusted by age and gender accordingly. *P *< 0.05 was considered statistically significant.

## Results

The demographics of the cases and controls enrolled in this study are shown in Table 1. There were no significant differences in the mean age and sex distribution between cases and controls, suggesting that the matching based on these two variables was adequate. There was no evidence of a deviation from Hardy-Weinberg equilibrium among the case or control subjects. The mean age was 65 years (range, 34 – 80 years) for the case patients and 71 years (range, 32 – 80 years) for the control subjects (Table 1).

**Figure 1:**
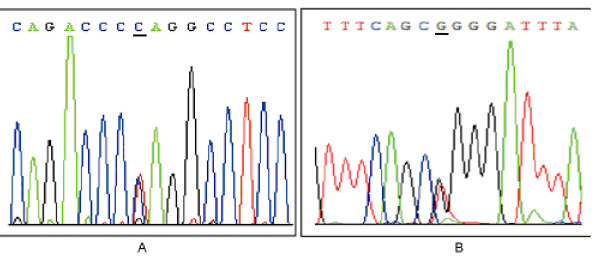
**Sequencing results for each of the PCR products from -149 C>T (A) and -579 G>T (B)**. Sequencing results for each of the PCR products from -149 C>T (A) and -579 G>T (B), the SNP sites are as indicated by the arrowhead. These results were completely matched to corresponding results deriving from PCR-RFLP by agarose gel genotyping method.

All patients and controls were successfully genotyped for the *DNMT3B *polymorphism. The genotyping by PCR-RFLP analysis was completely confirmed by DNA sequencing analysis, and the results of PCR-RFLP genotyping and sequencing analysis were also 100% concordant (Fig. 1). The distribution of the 149C>T and the 579G>T polymorphism of *DNMT3B *were in Hardy-Weinberg equilibrium. The distributions of *DNMT3B *149C>T and 579G>T genotypes among controls and cases are shown in Table 2. The allele frequency of 149C>T among patients and controls was 0.73% versus 0.65%, respectively. The allele frequency of 579G>T for patients and controls was 6.57% versus 11.53%, respectively. No significant deviation was observed for the genotype distributions of 149C>T polymorphisms between colorectal cancer cases and controls. However, the distributions of 579G>T genotypes in the colorectal cancer group (GG 0%, GT 13.14%, TT 86.86) were significantly different from those among the controls (GG 1.95%, GT 19.15%, TT 78.90%; 0.01 < P < 0.05).

**Table 2:** *DNMT3B *genotypes and allele frequency among controls and cases

	-149 C > T genotype				-579G.>T genotype			
		
	CC	CT	TT	C allele^b^ frequency (%)	GG	GT	TT	G allele^c ^ frequency (%)
Control	0(0.00)	4(1.30)	304(98.70)	0.65	6(1.95)	59(19.15)	243(78.9)	11.53
Case	0(0.00)	2(1.46)	135(98.54)	0.73	0(0.00)	18(13.14)	119(86.86)	6.57

^a^Numbers in parentheses, percentage.

^b^*p *> 0.05

^c^0.01 <*p *< 0.05

The colorectal cancer risk related to the *DNMT3B *149C>T and 579G>T genotypes were shown in table 3. ORs and their 95% CIs were calculated using the more common homozygous variant genotype as the reference group (149TT and 579 TT genotypes, respectively). Because of low prevalence of homozygous wild-type genotype, we combined this genotype with heterozygous genotype into one group and compared it with the reference group. As compared with the reference group, the combined 149CC and CT genotype, were associated with non-significant increased of colorectal cancer (OR = 1.13; 95% CI = 0.20–6.22). However, as compared with the reference group, the combined 579 GG and GT genotype, were associated with significant decreased of colorectal cancer (OR = 0.57 95% CI = 0.32–1.00). Then we stratified the results by age, patients and controls were found to be a little different with respect to the genotype distribution. Our data thus reveal that the presence of 579 G>T rather than 149 C>T exists significance likelihood of carcinogenesis for colorectal cancer patients, at least in a Chinese population. The results didn't show that 597 G>T is associated with the onset age of CRC in this study.

**Table 3:** Crude and adjusted ORs for colorectal cancer associated with *DNMT3B *genotypes

Genotypes	Cases
-149 T>C genotype	
TT (reference)	1.00
TC +CC crude	1.13 (0.20–6.22)
	
-579 G.>T genotype	
TT (reference)	1.00
GT + GG crude	0.57(0.32–1.00)

## Discussion

Colorectal cancer (CRC) is one of the most common cancers, with an estimated worldwide incidence of more than 570,000 new cases per year [29]. Despite recent surgical, radiotherapy and chemotherapy advances in the treatment protocol, the long-term survival of CRC patients still remains at approximately 50% for the past three decades. Early detection among susceptible populations has been advocated to decrease both the morbidity and the mortality of CRC, but searching for a useful stratification marker to predict its genetic susceptibility still would appear to impose a major challenge for CRC researchers.

DNA methylation has been reported to play a significant role in the development and progression of various cancers. In such cases, DNA methylation is typically mediated by DNA methyltransferases (DNMTs), specifically *DNMT1*, *DNMT3A *and *DNMT3B*. *DNMT1 *is considered as a maintenance DNA methyltransferase due to its ability to preferentially methylate hemimethylated DNA subsequent to DNA replication [30,31]. *DNMT3A *and *DNMT3B *function as *de novo *methyltransferases, which reportedly methylate unmethylated and hemimethylated DNA with equal efficiencies [32].

A single nucleotide polymorphism (SNP) of the *DNMT3B *promoter polymorphism -149 C>T, have recently been shown increase susceptibility of an individual to lung cancer [2], breast cancer [23] and significantly associated with increased age-associated risk in HNPCC families [24], but not to head and neck squamous cell carcinoma in Taiwanese [33], HCC [34] and gastric cardiac adenocarcinoma in Chinese [35], and gastric cancer in a Japanese population [36]. Another single nucleotide polymorphism (SNP) of the *DNMT3B *promoter 579G > T is from exon 1B transcription start site of *DNMT3B*. Although it is supposed probably affect the gene function [27], the 579 G>T polymorphisms did not significantly change the promoter activity of DNMT3B by luciferase assay [26]. However, studies recent been shown decrease susceptibility of an individual to lung cancer [2] and colon cancer [27]. These data suggested that DNMT3B promoter 579G > T polymorphism can be used a risk factor of cancer to evaluate the population susceptible to tumors. Some other study haven't shown association between polymorphism of 579G > T and head and neck squamous cell carcinoma [33], and esophagus cancer [37].

In the current study, we investigated the influence of *DNMT3B *polymorphisms -149 C>T and -579G > T on the risk of colorectal cancer in a hospital-based case-control study in a Chinese population. For -149 C>T, CC genotype was not detectable in both CRC patients and controls, while the T allele was predominant just like in a Taiwanese population [33] and a Japanese population [36]. There was no significant difference between CRC patients and control in -149 C>T of *DNMT3B *polymorphisms. These data implied that, an individual with the -149C>T polymorphism has not an increased susceptibility to CRC cancer in the study Chinese population. However, carriers with 579G allele were at decreased risk of CRC as compared with individuals having 579T alleles. This finding suggests that the 579G>T polymorphism in the *DNMT3B *gene could be used as a marker of genetic susceptibility to CRC cancer.

Our findings are consistent with those of studies, which showed that individuals carrying the G allele have a significantly lower risk of developing adenocarcinoma of the lung cancer [2] and colon cancer [27]. Carriers with the G allele in the *DNMT3B *gene were found to have a decreased risk of colon cancer compared with individuals with the T allele. These findings implied that the *DNMT3B *polymorphism might operate in a tissue-specific manner, especially in colorectal tissue. However, this finding needs to be confirmed by a larger study. The present result, and those from lung and colon cancer which are the only published data on association between *DNMT3B *SNP and cancer development, can show the different possible roles of *DNMT3B *in different cell types. Since the different splice variants of *DNMT3B *which may alter catalytic activity are expressed in a tissue specific manner [10,38-40] and repression of *DNMT3B *activity does not result in the re-expression of all hypermethylated tumor suppressor genes in some cell system [41-44], it is therefore important to explore the complex interplay of DNMTs in different tumor types. Since genetic polymorphisms often vary between ethnic groups, further studies are needed to clarify the association of the *DNMT3B *polymorphism with colorectal cancer in diverse ethnic populations. Future studies of other *DNMT3B *sequence variants and their biologic function are also needed to understand the role of *DNMT3B *polymorphisms in determining the risk of cancer.

## Conclusion

The *DNMT3b *-579G>T polymorphism may contribute to the genetic susceptibility to colorectal cancer. Carriers with the G allele in the *DNMT3B *gene were found to have a decreased risk of colorectal cancer compared with individuals with the T allele. However, the relative distribution of -149C>T *DNMT3B *SNP among a Chinese population can not be used as a stratification marker to predict an individual's susceptibility to colorectal cancer.

## Competing interests

The authors declare that they have no competing interests.

## Authors' contributions

HF carried out *DNMT3B *-579G>T polymorphism analysis and wrote the manuscript, FZ collected the samples and patient's clinical data. DSL and JBH performed -149C>T DNMT3B SNP research and analyzed the data. ZJZ contributed to new reagents/analytic tools. All authors read and approved the final manuscript.
